# Efficacy and safety of sodium-glucose cotransporter 2 inhibitors in the treatment of diabetic kidney disease: a meta-analysis

**DOI:** 10.3389/fendo.2025.1596888

**Published:** 2026-01-27

**Authors:** Yingbo Guo, Wenfeng Gao, Shichao Li, Xiaona An, Zhongjie Liu

**Affiliations:** 1 Department of Nephrology, Dongfang Hospital, Beijing University of Chinese Medicine, Beijing, China; 2 Department of Urology, Dongzhimen Hospital, Beijing University of Chinese Medicine, Beijing, China; 3 Department of Nephrology, Dongzhimen Hospital, Beijing University of Chinese Medicine, Beijing, China

**Keywords:** efficacy, diabetic kidney disease, sodium-glucose cotransporter 2, estimated glomerular filtration, meta-analysis

## Abstract

**Background:**

Diabetic kidney disease is a major cause of end-stage renal disease. Herein, we aimed to assess the efficacy and safety of sodium-glucose cotransporter 2 (SGLT2) inhibitors in patients with diabetic kidney disease.

**Methods:**

PubMed, Embase, and Web of Science databases were searched for eligible randomized clinical trials (RCTs) published up to July 2024. Effect sizes were summarized as risk ratios (RR) or weighted mean differences (WMD) with 95% confidence intervals (CI). Statistical analyses were performed using Stata.

**Results:**

Fifteen studies (24463 patients) were included in the meta-analysis. The results of the meta-analysis showed that compared with the control group, SGLT2 inhibitor intervention could reduce the estimated glomerular filtration rate (WMD=−2.47; 95% CI: −3.18, −1.76), systolic blood pressure (WMD=−4.09; 95% CI: −4.97 to -3.21), diastolic blood pressure (WMD=−2.47; 95% CI: −3.06 to −1.88), and glycated hemoglobin (WMD=−0.27; 95% CI: −0.38, −0.17). Moreover, there was no significant difference between the SGLT2 inhibitor and control groups in terms of the incidence of overall adverse event, urinary tract infection, bone fracture and hypoglycemia. However, the incidence of genital infection and diabetic ketoacidosis in the SGLT2 inhibitor group was higher than that in the control group.

**Conclusion:**

Our study confirms the beneficial effects in diabetic kidney disease patients, while also demonstrating a higher risk of genital infections and diabetic ketoacidosis in the SGLT2 inhibitor group compared to controls.

## Introduction

Diabetic kidney disease, also known as diabetic nephropathy, is characterized by the chronic loss of kidney function in individuals with diabetes mellitus (DM). Diabetic kidney disease is a major cause of chronic kidney disease (CKD) and end-stage renal disease (ESRD) worldwide (1–4). Urine protein loss due to a glomerular injury can worsen and cause low serum albumin levels, resulting in widespread edema and nephrotic syndrome (5). Patients with microalbuminuria develop macroalbuminuria, ultimately resulting in ESRD (5). Furthermore, glomerular filtration rate (GFR), a key predictor of renal insufficiency, may gradually decline in individuals with normal albumin excretion or progressive low-level microalbuminuria (6).

It is well-known that sodium-glucose cotransporter 2 (SGLT2) inhibitors primarily function at SGLT2, distributed in the anterior portion of the proximal tubule, to decrease renal tubular sodium reabsorption, thereby increasing the excretion of urinary glucose and sodium to minimize blood glucose and glycosylated hemoglobin levels (7, 8). Given that the action of SGLT2 inhibitors does not rely on insulin, these agents have been deemed valuable at all stages of type 2 DM (T2DM), exhibiting a low potential for hypoglycemia during treatment and possibly reducing weight, blood pressure, uric acid levels, and other symptoms (9–11). The findings of various clinical trials indicate that SGLT2 inhibitors may improve cardiovascular and renal outcomes in patients at high cardiovascular risk in the treatment of T2DM, owing to their pleiotropic nature (12, 13). Therefore, in the present study, we aimed to evaluate the efficacy and safety of SGLT2 inhibitors in patients with diabetic kidney disease by performing a meta-analysis to summarize and update the evidence regarding the impact of SGLT2 inhibitors on patients with diabetic kidney disease.

## Methods

The review protocol has been registered in the International Platform of Registered Systematic Review and Meta-Analysis Protocols (PROSPERO registration number: CRD42024518979).

### Data sources

The online database was indexed in PubMed, the Cochrane Library, and the Embase Database and searched for randomized controlled trials (RCTs) linked to SGLT2 inhibitor therapy for diabetic nephropathy. The search was conducted up to July 2024. The following keywords and medical subject terms (MeSH) were searched: sodium-glucose cotransporter 2 inhibitor, canagliflozin, dapagliflozin, empagliflozin, ipragliflozin, ertugliflozin, diabetic nephropathy, diabetic kidney disease, diabetes mellitus, and chronic kidney diseases. The search was not restricted by language. Furthermore, paper-based documents and screened relevant reviews and respective references were manually searched to obtain additional studies that can be used for meta-analysis. The detailed search strategy is described in **Supplementary Material 1**.

To formulate strict guidelines for study inclusion and exclusion, the selected literature must be based on the following criteria. (1) the type of study is RCT; (2) scientifically diagnosed diabetic kidney disease; (3) intervention measures: the therapeutic group was taking SGLT2 inhibitors; there is no dose and medication limitation; the control group received a placebo. (4) Outcome indicators included estimated GFR (eGFR), systolic blood pressure (SBP), diastolic blood pressure (DBP), glycated hemoglobin (HbA1c) and adverse events (AEs).

Exclusion criteria were as follows: (1) unrandomized clinical trials; (2) kidney damage caused by conditions other than type 1 or type 2 diabetes; (3) removal of non-authoritative literature, such as reviews, letters, and comments; (4) repetitive/duplicate reports or the same demographic data used in several surveys (either the most recent study or one with complete detail was used, excluding the remainder).

### Data extraction and evaluation of the quality

Two authors independently extracted relevant data from the included literature, and the extracted content included research characteristics (year of publication and first author), participants (sample size and age), intervention measures, performance metrics (eGFR, SBP, DBP, HbA1c and AEs), and duration of follow-up after the intervention.

The Cochrane risk-of-bias tool was used to measure the efficiency of included RCTs.

After completing data extraction and quality evaluation, the related forms were interactively checked. In the case of non-consensus regarding data extraction and quality evaluation process, discussions were initiated, and a third investigator was consulted to achieve a consistent outcome.

### Statistical analysis

Stata 15.0 was used to perform statistical analysis. The weighted mean difference (WMD) was used for continuous variables, the risk ratio (RR) was used for dichotomous variables, and all variables were expressed as 95% confidence intervals (CI). We used I^2^ statistics to determine the RCT heterogeneity. If I^2^ was <50%, heterogeneity was considered appropriate, and a fixed-effect model was employed to evaluate the results. If I^2^ was >50%, subgroup or sensitivity analysis was performed to address heterogeneity. If the variability remained at >50%, a random-effects model was used to evaluate the results. Egger’s test was used to detect publication bias. Meta-regression analysis was performed to evaluate the potential source of heterogeneity among studies. Moreover, a leave-one-out sensitivity analysis was conducted to assess the robustness of the findings by sequentially excluding individual studies.

## Results

**Figure 1:**
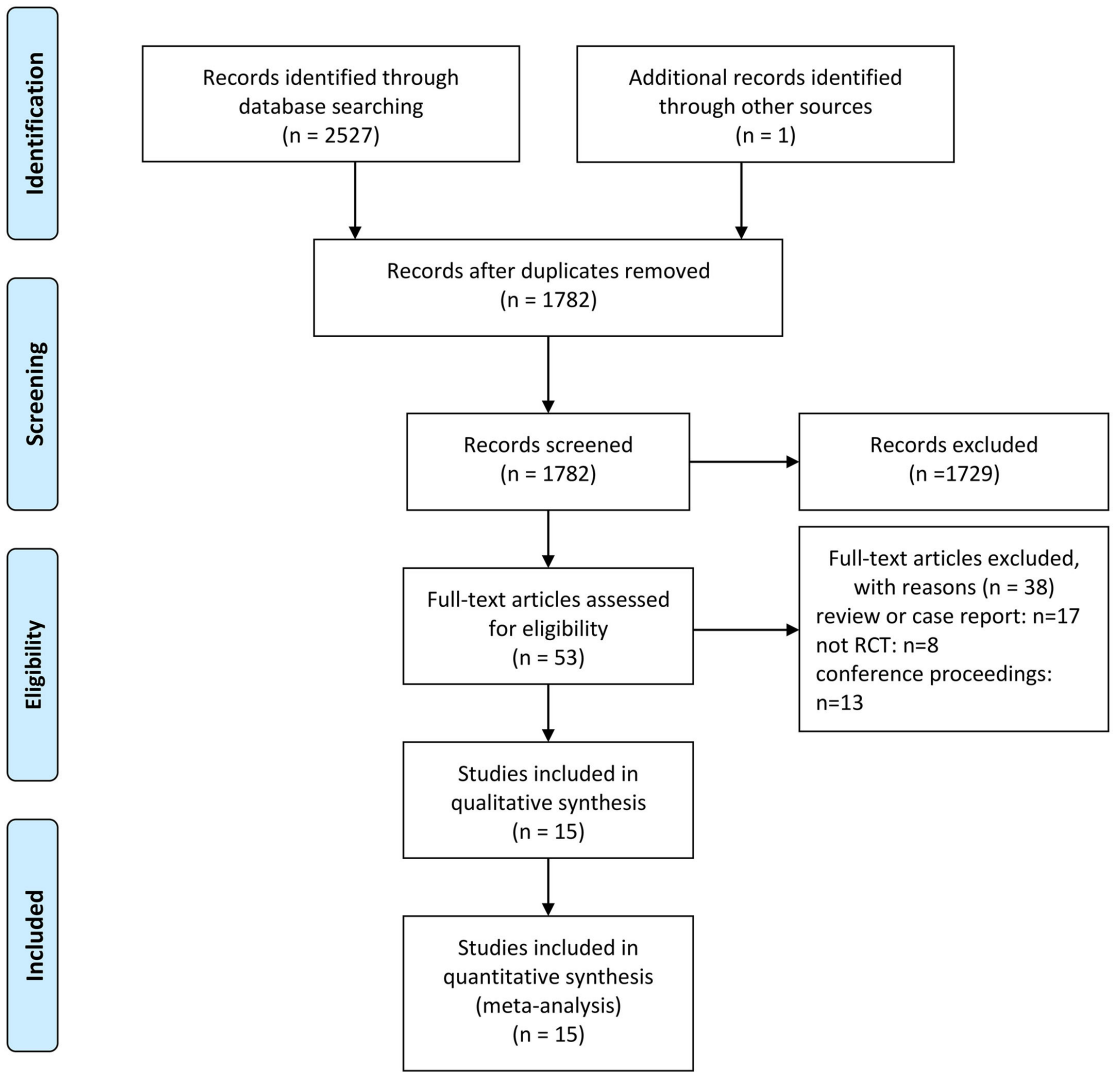
Flow diagram of study selection.

**Figure 2:**
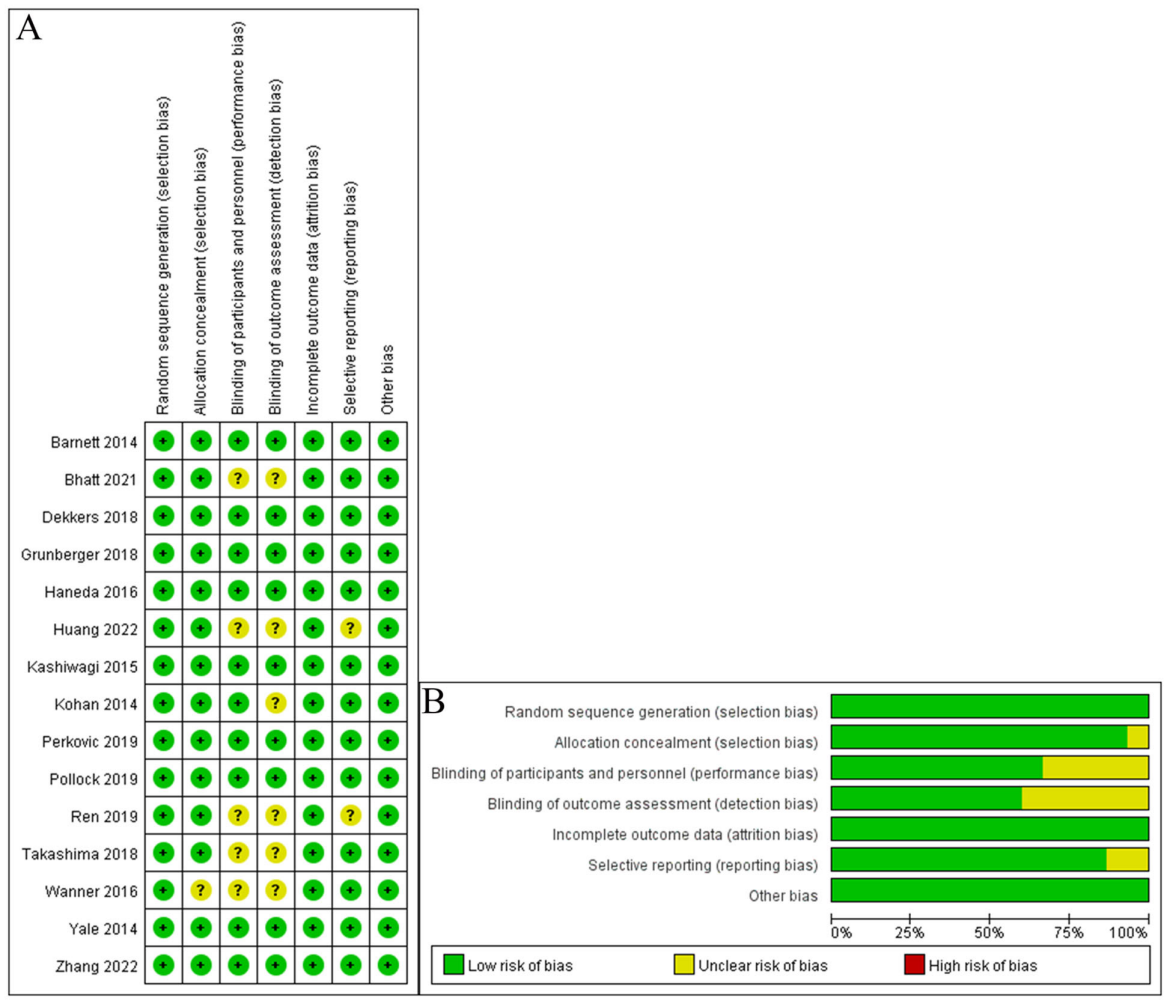
Quality assessments of the included RCTs articles. **(A)** Risk of bias graph. **(B)** Risk of bias summary.

A total of 2528 relevant studies in Chinese and English were identified, of which 746 were omitted owing to duplication of literature. Overall, 1729 studies were omitted from the reading of titles and abstracts. Subsequently, the entire text was checked based on inclusion and exclusion requirements, resulting in the exclusion of 38 sources. Finally, 15 relevant studies were included, comprising 24463 patients (14–28). A flow map of literature selection and screening is shown in **Figure 1**. The length of the illness varied from 9.5 to 16.9 years. With the exception of two studies by Takashima et al. (25) and Bhatt et al. (15), the baseline HbA1c level of patients was >8%. The basic features of included studies are summarized in **Table 1**. Most studies provide a comprehensive description of the random sequence generation process and implement blinding for participants, personnel, and outcome assessment. Overall, the risk of selective reporting is low. The results of the risk-of-bias graph are shown in **Figure 2**.

**Table 1:** Characteristics of included studies.

Study	Intervention	N	Mean age	Mean baseline HbA1c (%)	Mean baseline eGFR (ml/min/1.73 m2)	Mean duration of diabetes (years)	Duration of intervention (weeks)
Barnett 2014 (14)	Empagliflozin VS. placebo	290	62.6 ± 8.3	8.03 ± 0.79	/	/	52 weeks
Yale 2014 (27)	Canagliflozin vs. placebo	269	68.5 ± 8.3	8.0 ± 0.9	39.4 ± 6.9	16.3	104 weeks
Kohan 2014 (22)	Dapagliflozin vs. placebo	252	67	13.5	8.3 ± 1.04	16.9	104 weeks
Kashiwagi 2015 (21)	Ipragliflozin vs. placebo	165	64	8.09 ± 1.337	60.5 ± 13.1	9.5	24 weeks
Wanner 2016 (26)	Empagliflozin VS. placebo	7018	<65	>8	/	/	192 weeks
Haneda 2016 (18)	Luseogliflozin vs. placebo	150	>65	>8	/	11.1	24 weeks
Dekkers 2018 (16)	Dapagliflozin vs. placebo	220	66.28	8.22	38 ± 5	/	24 weeks
Takashima 2018 (25)	Canagliflozin vs. placebo	42	65.05	7.4	56.25	/	52 weeks
Hujun 2019 (20)	Dapagliflozin vs. placebo	60	<65	>8	/	10.9	12 weeks
Grunberger 2018 (17)	Ertugliflozin vs. placebo	467	67.3 ± 8.6	8.2 ± 0.9	46.6 ± 8.8	14.2	52 weeks
Perkovic 2019 (23)	Canagliflozin vs. placebo	4401	63.0 ± 9.2	8.3 ± 1.3	56.2 ± 18.2	/	136 weeks
Pollock 2019 (24)	Canagliflozin vs. placebo	293	64.7 ± 8.5	8.51	48.94	/	24 weeks
Bhatt 2021 (15)	Sotagliflozin vs. placebo	10584	69 (63–74)	>7	44.7 (37.0–51.5)	/	64 weeks
Zhang 2022 (28)	Canagliflozin vs. placebo	132	54.75	8.66	97.07	5.43	24 weeks
Huang 2022 (19)	Dapagliflozin vs. placebo	120	55.94	9.335	/	9.845	13.3 weeks

### eGFR

**Figure 3:**
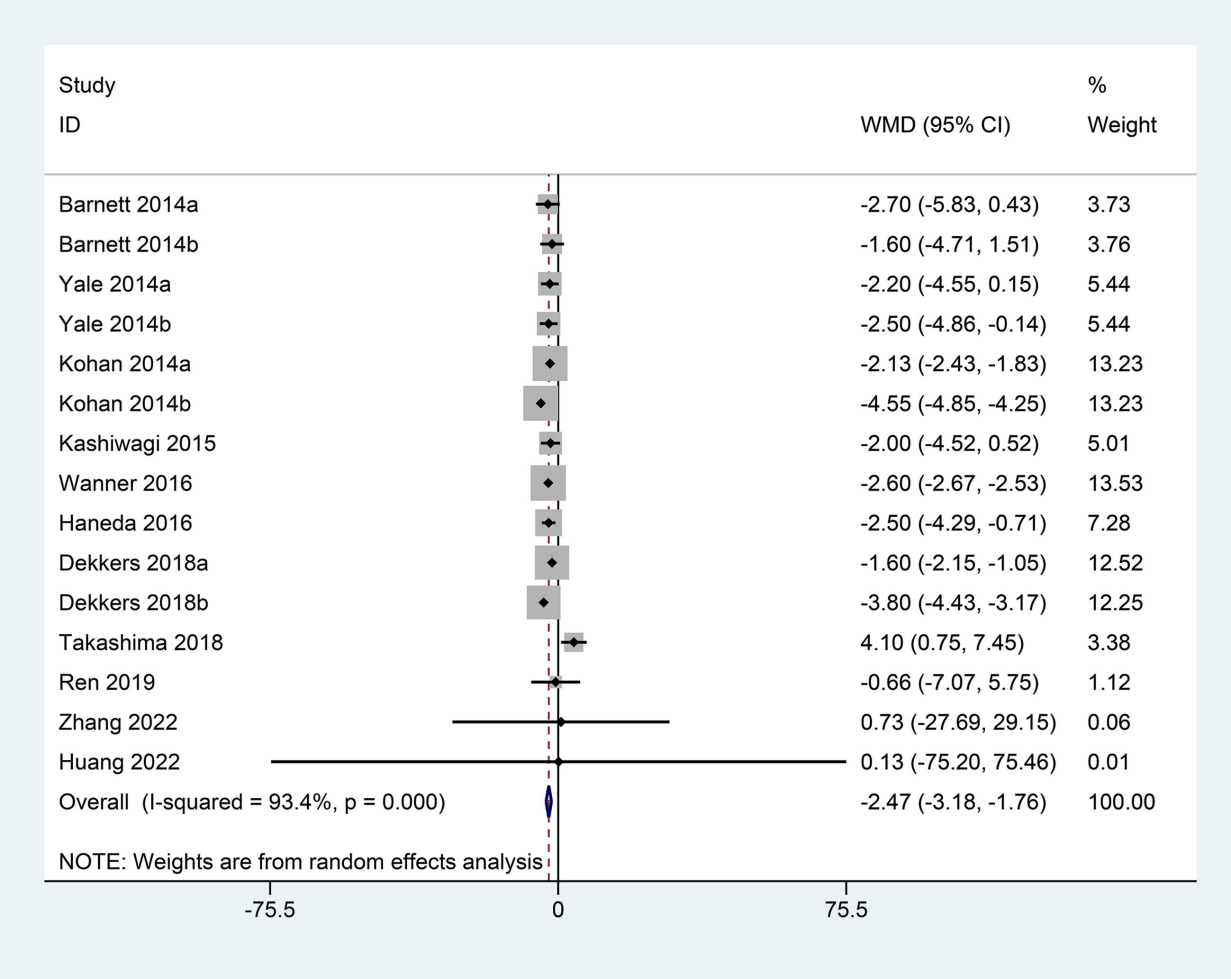
Forest plots of eGFR for SGLT2 inhibitors versus placebo in patients with diabetic kidney disease.

Eleven studies reported changes in eGFR from the baseline. Owing to the high heterogeneity (I^2^ = 93.4%, P<0.001), a random-effects model was used for the analysis. Based on the pooled analysis, eGFR was significantly reduced in the SGLT2 inhibitor group when compared with that in the control group (WMD=−2.47; 95% CI: −3.18, −1.76) (**Figure 3**).

### SBP

**Figure 4:**
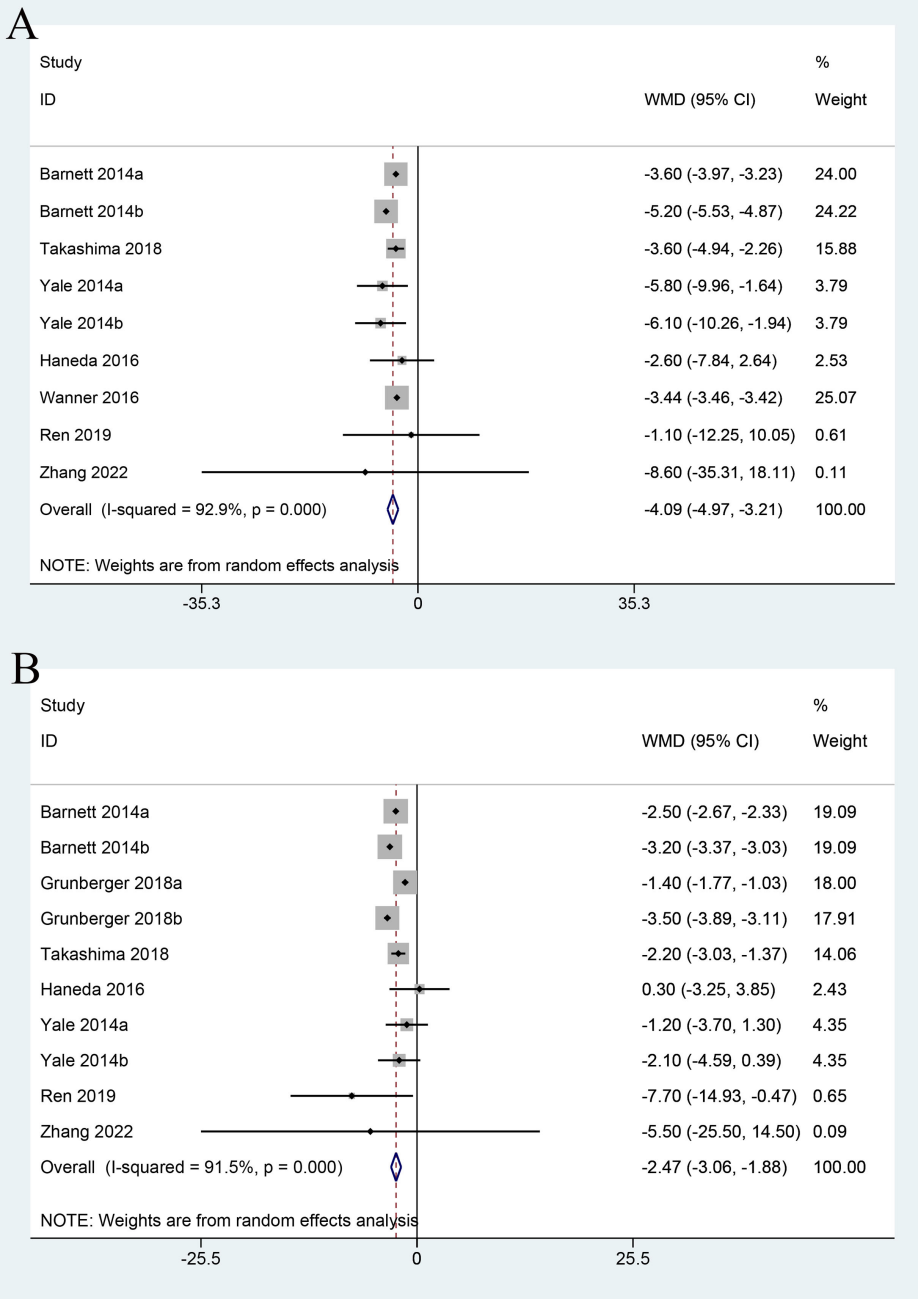
Forest plots of SBP **(A)** and DBP **(B)** for SGLT2 inhibitors versus placebo in patients with diabetic kidney disease.

Seven studies evaluated the SBP. We noted significant heterogeneity among studies (I^2^ = 92.9%, P<0.001); therefore, a random-effects model was employed. Compared with the control group, SBP was significantly reduced in the SGLT2 inhibitor group when the intervention duration was ≤26 weeks (WMD=−4.09; 95% CI: −4.97 to -3.21) (**Figure 4A**).

### DBP

Seven studies examined the effect of SGLT2 inhibitors on DBP, and significant heterogeneity was observed among studies (I^2^ = 91.5%, P<0.001), followed by the application of a random-effects model. Compared with the control group, the SCLT-2 group exhibited a significant reduction in DBP when the intervention duration was ≤26 weeks (WMD=−2.47; 95% CI: −3.06 to −1.88) (**Figure 4B**).

### HbA1c

**Figure 5:**
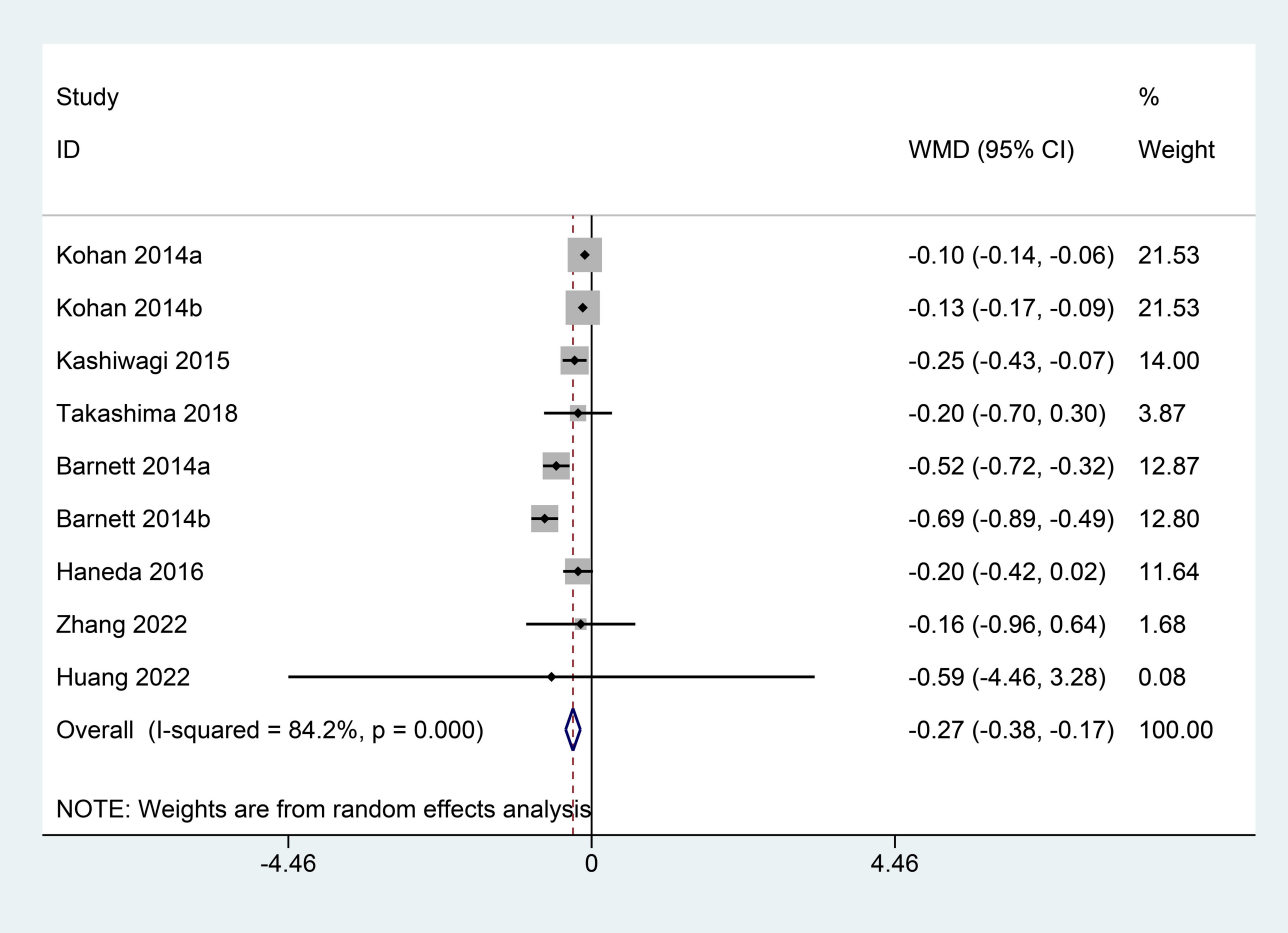
Forest plots of HbA1c for SGLT2 inhibitors versus placebo in patients with diabetic kidney disease.

Seven studies examined the effect of SGLT2 inhibitors on HbA1c. Owing to the high heterogeneity (I^2^ = 84.2%, P<0.001), a random-effects model was used for the analysis. Based on the pooled analysis, HbA1c was significantly reduced in the SGLT2 inhibitor group when compared with that in the control group (WMD=−0.27; 95% CI: −0.38, −0.17) (**Figure 5**).

### AEs

**Figure 6:**
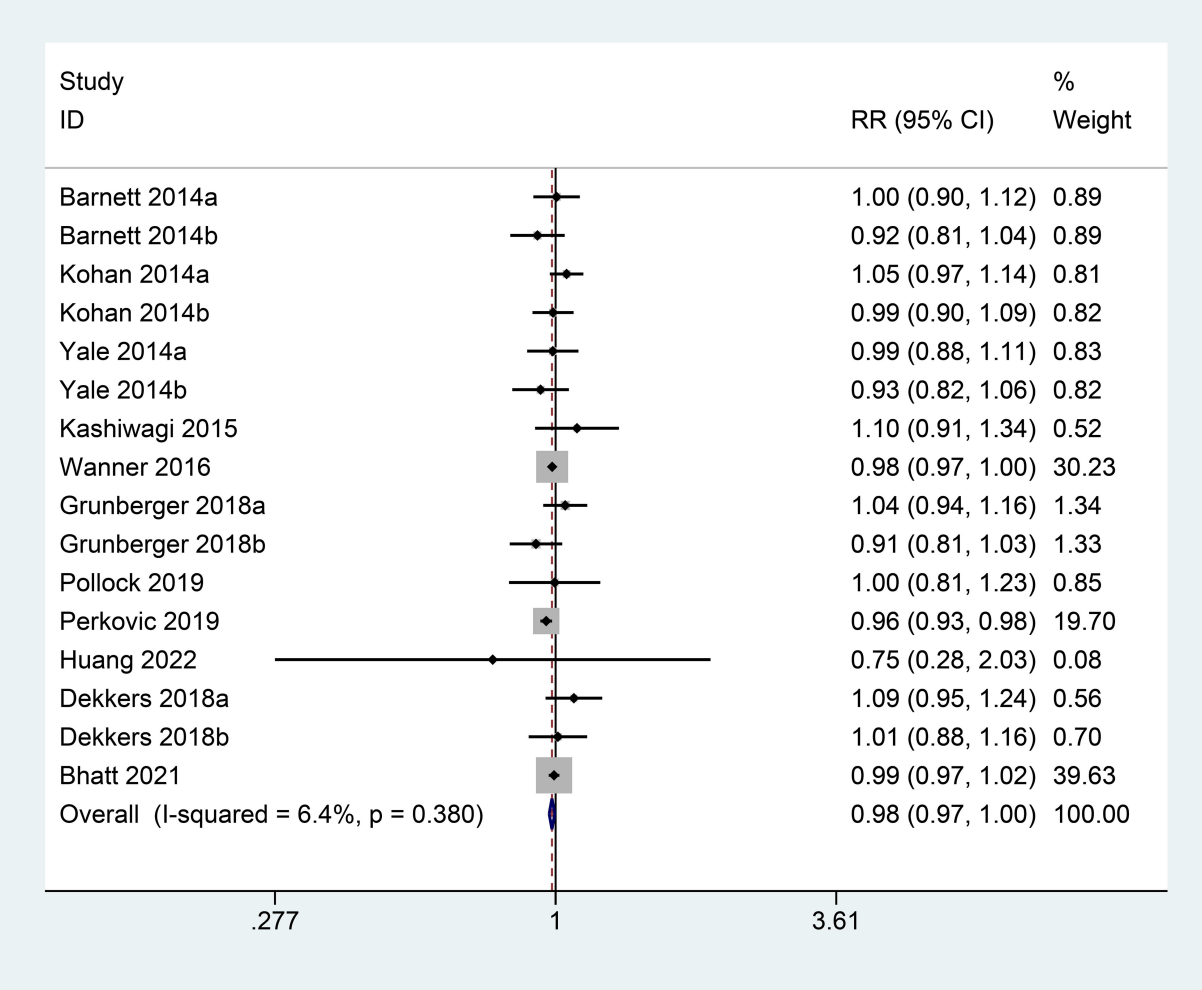
Forest plots of AEs for SGLT2 inhibitors versus placebo in patients with diabetic kidney disease.

Eleven of the included studies reported AEs. There was no evidence of heterogeneity among these studies (I^2^ = 6.4%, P = 0.380); therefore, a fixed-effects model was used. Based on the results, there was no significant difference in the total incidence of AE between SGLT2 inhibitor group and control group (RR = 0.98; 95% CI: 0.97 to 1.00) (**Figure 6**).

**Figure 7:**
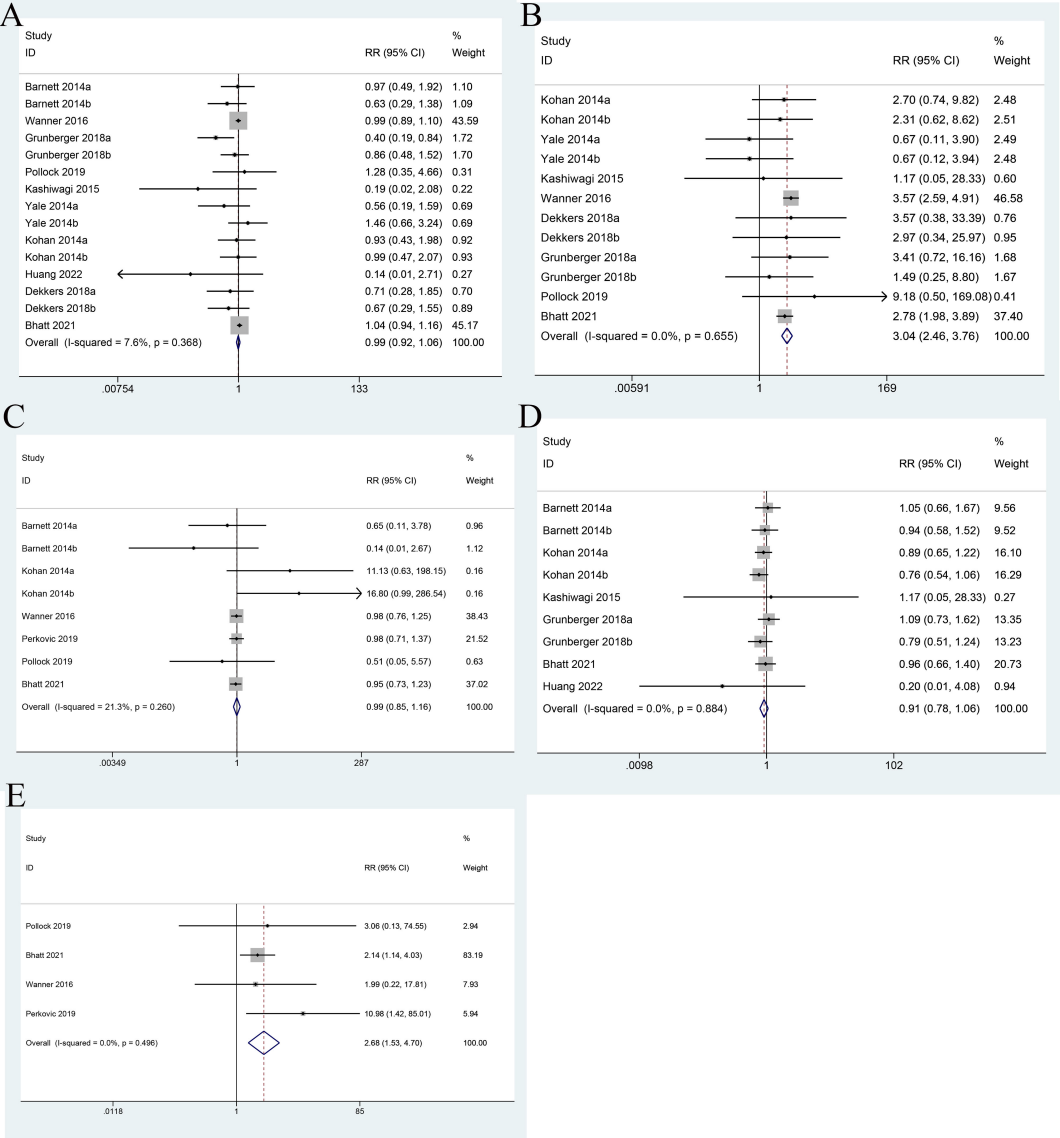
Forest plots of urinary tract infection **(A)**, genital infection **(B)**, bone fracture **(C)**, hypoglycemia **(D)**, and diabetic ketoacidosis **(E)** for SGLT2 inhibitors versus placebo in patients with diabetic kidney disease.

Ten studies reported the incidence of urinary tract infection. There was no heterogeneity between the studies; therefore, a fixed-effects model was used (I^2^ = 7.6%, P = 0.368). The incidence of urinary tract infection did not differ between the SGLT2 inhibitor group and the control group (RR = 0.99; 95% CI: 0.92 to 1.06) (**Figure 7A**).

Eight studies reported the incidence of genital infection. There was no heterogeneity between the studies; therefore, a fixed-effects model was used (I^2^ = 0.00%, P = 0.655). The result showed that the incidence of genital infection in the SGLT2 inhibitor group was higher than that in the control group (RR = 3.04; 95% CI: 2.46 to 3.76) (**Figure 7B**).

Six studies reported the incidence of bone fracture. There was no heterogeneity between the studies; therefore, a fixed-effects model was used (I^2^ = 21.3%, P = 0.20). There was no significant difference in the incidence of bone fracture between SGLT2 inhibitor group and control group (RR = 0.99; 95% CI: 0.85 to 1.16) (**Figure 7C**).

Six studies reported the incidence of hypoglycemia. There was no heterogeneity between the studies; therefore, a fixed-effects model was used (I^2^ = 0.0%, P = 0.884). There was no significant difference in the incidence of hypoglycemia between SGLT2 inhibitor group and control group (RR = 0.91; 95% CI: 0.78 to 1.06) (**Figure 7D**).

Four studies reported the incidence of diabetic ketoacidosis. There was no heterogeneity between the studies; therefore, a fixed-effects model was used (I^2^ = 0.00%, P = 0.496). The result showed that the incidence of diabetic ketoacidosis in the SGLT2 inhibitor group was higher than that in the control group (RR = 2.68; 95% CI: 1.53 to 4.70) (**Figure 7E**).

### Meta-regression

To explore potential sources of heterogeneity, a meta-regression analysis was performed on the outcomes (eGFR, SBP, DBP, and HbA1c). The results indicated that the type of drug, sample size, publication year, and follow-up duration could not explain the observed heterogeneity in eGFR, SBP, and DBP (P>0.05, **Table 2**). However, for HbA1c, sample size (P = 0.017) and follow-up duration (P = 0.027) significantly contributed to the heterogeneity.

**Table 2:** Results of meta-regression analysis.

Outcomes	Coef.	Std. Err.	z	P>│z│	[95% Conf. Interval]
eGFR					
Type of Drug	-0.5539943	0.558288	-0.99	0.321	(-1.648219, 0.5402302)
Sample size	-0.0003379	0.0005307	-0.64	0.524	(-0.0013781, 0.0007023)
Publication year	0.4937251	0.4537555	1.09	0.277	(-0.3956193, 1.383069)
Follow-up	0.0047758	0.0236753	0.2	0.84	(-0.0416269, 0.0511784)
SBP					
Type of Drug	0.2473853	1.373909	0.18	0.857	(-2.445427, 2.940198)
Sample size	0.0007791	0.000726	1.07	0.283	(-0.0006437, 0.002202)
Publication year	0.2073946	0.3764145	0.55	0.582	(-0.5303642, 0.9451534)
Follow-up	-0.0328584	0.0321941	-1.02	0.307	(-0.0959578, 0.0302409)
DBP					
Type of Drug	1.030132	0.7636155	1.35	0.177	(-0.4665265, 2.526791)
Sample size	0.0026365	0.0037474	0.7	0.482	(-0.0047082, 0.0099813)
Publication year	-0.28589	0.3322243	-0.86	0.389	(-0.9370377, 0.3652576)
Follow-up	-0.0103947	0.0293708	-0.35	0.723	(-0.0679605, 0.047171)
HbA1c					
Type of Drug	-0.0293629	0.045515	-0.65	0.519	(-0.1185707, 0.0598448)
Sample size	-0.003258	0.0013629	-2.39	0.017	(-0.0059292, -0.0005869)
Publication year	-0.0217008	0.0647169	-0.34	0.737	(-0.1485435, 0.1051419)
Follow-up	0.0045354	0.0020562	2.21	0.027	(0.0005052, 0.0085655)

### Publication bias and sensitivity analyses

Egger test of each outcome showed P>0.05, indicating that there was no publication bias (**Supplementary Figure S1**). Sensitivity analysis showed that when each study was omitted in turn, the pooled estimates did not change significantly (**Supplementary Figure S2**), indicating that the result were robust.

## Discussions

In the United States, approximately 40% of individuals with type 2 diabetes present with CKD, and type 2 diabetes is generally regarded as the primary cause of ESRD (29). Diabetic kidney disease is a chronic complication characterized by microvascular lesions. In addition to persistent hyperglycemia, the incidence and development of diabetic kidney disease are frequently associated with other risk factors, such as obesity, hypertension, dyslipidemia, and genetic susceptibility (1, 30). SGLT2 antagonists can block sodium-glucose reabsorption in proximal renal tubules, inducing osmotic diuresis and natriuresis. In both the EMPA-reg and CANVAS Program trials, SGLT2 inhibitors (empagliflozin and canagliflozin) were found to reduce proteinuria progression (31, 32). Therefore, our meta-analysis builds on these findings by incorporating the most recent evidence from large-scale trials, including DAPA-CKD and EMPA-KIDNEY, to provide updated insights into the efficacy and safety of SGLT2 inhibitors in DKD.

This meta-analysis advances existing knowledge by incorporating the most recent RCTs published up to July 2024, including landmark studies such as DAPA-CKD and EMPA-KIDNEY, thereby expanding the evidence base with contemporary data. With 24,463 patients included, this is one of the largest meta-analyses to date evaluating both renal and metabolic outcomes in diabetic kidney disease, enabling precise effect size estimation and robust subgroup analyses. Notably, our findings refine the safety profile of SGLT2 inhibitors by quantifying a statistically significant increase in genital infections (RR = 3.04) and diabetic ketoacidosis (RR = 2.68) in this population—a critical consideration given the mechanistic link between glucosuria and genital infection risk, as well as the insulin-independent action of SGLT2 inhibitors potentially exacerbating ketogenesis.

Considering blood pressure, weight loss combined with osmotic diuretics and the reduction of overall sodium in the body will potentially reduce BP, approximately 4–6 mmHg for SBP and 1–2 mmHg for DBP. Based on the findings of the present study, SGLT2 inhibitors were substantially more effective than placebo, and the effect of SGLT2 inhibitors on blood pressure reduction was impaired by renal activity, period of drug use, baseline HbA1c level, and body mass index. It is well-documented that SGLT2 inhibitors can effectively reduce blood pressure and lower SBP, which could be attributed to an early SGLT2 inhibitor triggering osmotic diuresis and a drop in blood volume due to urinary sodium excretion, increased urinary glucose and sodium excretion, weight loss, and a 6-month blood pressure impact (33).

SGLT2 inhibitors can induce a decrease in eGFR. The decreased eGFR may be attributed to the ability of SGLT2 inhibitors to reduce vasodilation of afferent arterioles by increasing sodium transport to the macula densa and restoring tubular glomerular feedback, which is the main pathophysiological cause underlying diabetic proteinuric nephropathy (34). However, studies have shown that the decline in eGFR can be completely reversed following drug discontinuation (35).

In terms of efficacy, there was no significant difference between the SGLT2 inhibitor and control groups concerning the incidence of overall adverse events, urinary tract infections, bone fractures, and hypoglycemia. This finding is consistent with previous studies. Xu et al. demonstrated that, compared to metformin, SGLT2 inhibitors do not increase the risk of urogenital infections (36). Deshpande et al. found no significant difference in the incidence of urinary tract infections when comparing SGLT2 inhibitors with placebo in patients (37). Wiviott et al. found that SGLT2 inhibitors are not associated with an increased risk of hypoglycemia (38). Meta-analyses and population-based studies of SGLT2 inhibitor therapy have largely not demonstrated an increased risk of fractures (39, 40). However, Our study found that the incidence of genital infections and diabetic ketoacidosis was higher in the SGLT2 inhibitor group than in the control group. These findings are consistent with previous literature reports. The DECLARE-TIMI 58 trial showed that users of SGLT2 inhibitors are more prone to developing genital fungal infections (38). Hamblin et al. found that SGLT2 inhibitor users are more likely to develop diabetic ketoacidosis during hospitalization compared to non-users (41). The increase in genital infections may be related to the mechanism of SGLT2 inhibitors, which inhibit renal tubular glucose reabsorption, increasing urinary glucose excretion and thus providing an environment conducive to bacterial and fungal growth (42). Although the incidence of diabetic ketoacidosis is low, its increased risk may be associated with SGLT2 inhibitors stimulating the release of glucagon, thereby increasing ketone body production (43). Despite the significant advantages of SGLT2 inhibitors in lowering blood glucose and improving cardiovascular outcomes, their potential adverse events, particularly genital infections and diabetic ketoacidosis, need to be carefully considered in clinical practice.

Nevertheless, the limitations of the present study must be acknowledged. Firstly, variations across RCTs in patient populations, drug types within each class, dosages, and follow-up durations may introduce heterogeneity, potentially affecting the meta-analysis results. Secondly, although a meta-regression analysis was conducted to explore sources of consistency, the origins of heterogeneity for outcomes such as eGFR, SBP, and DBP were not identified. Thirdly, there is a lack of long-term follow-up data. Fourthly, despite its clinical importance, the outcome of albuminuria could not be pooled due to insufficient reporting in most of the included RCTs and significant heterogeneity in the measurement and reporting of this outcome. This omission limits the scope of our renal efficacy assessment. Future research should focus on high-quality, long-term follow-up, rigorously designed RCTs with larger sample sizes.

In summary, our study corroborates the positive impact of SGLT2 inhibitors on estimated glomerular filtration rate, blood pressure, and glycated hemoglobin levels in patients with diabetic kidney disease. Notably, there were no significant differences between the SGLT2 inhibitor and control groups concerning the overall incidence of adverse events, including urinary tract infections, bone fractures, and hypoglycemia. However, the incidence of genital infections and diabetic ketoacidosis was higher in the SGLT2 inhibitor group compared to the control group. Therefore, clinicians should be vigilant for cases of euglycemic diabetic ketoacidosis in patients with diabetic kidney disease treated with SGLT2 inhibitors.

## Data Availability

The original contributions presented in the study are included in the article/**Supplementary Material**. Further inquiries can be directed to the corresponding author.

## Supplementary material

Supplementary Figure 1 Egger’s plots of eGFR **(A)**, SBP **(B)**, DBP **(C)**, HbA1c **(D)**, AEs **(E)**, urinary tract infection **(F)**, genital infection **(G)**, bone fracture **(H)**, hypoglycemia **(I)**, and diabetic ketoacidosis **(J)**. AEs, adverse events; DBP, diastolic blood pressure; eGFR, estimated glomerular filtration rate; SBP, systolic blood pressure.

Supplementary Figure 2 Sensitivity analysis of eGFR **(A)**, SBP **(B)**, DBP **(C)**, HbA1c **(D)**, and AEs **(E)**.
