# Supplementary figures and images for: Efficacy and safety of sodium-glucose cotransporter 2 inhibitors in the treatment of diabetic kidney disease: a meta-analysis
Source: Front Endocrinol (Lausanne). 2026 Jan 27;16:1596888. doi: 10.3389/fendo.2025.1596888 (PMC12886043; doi:10.3389/fendo.2025.1596888)

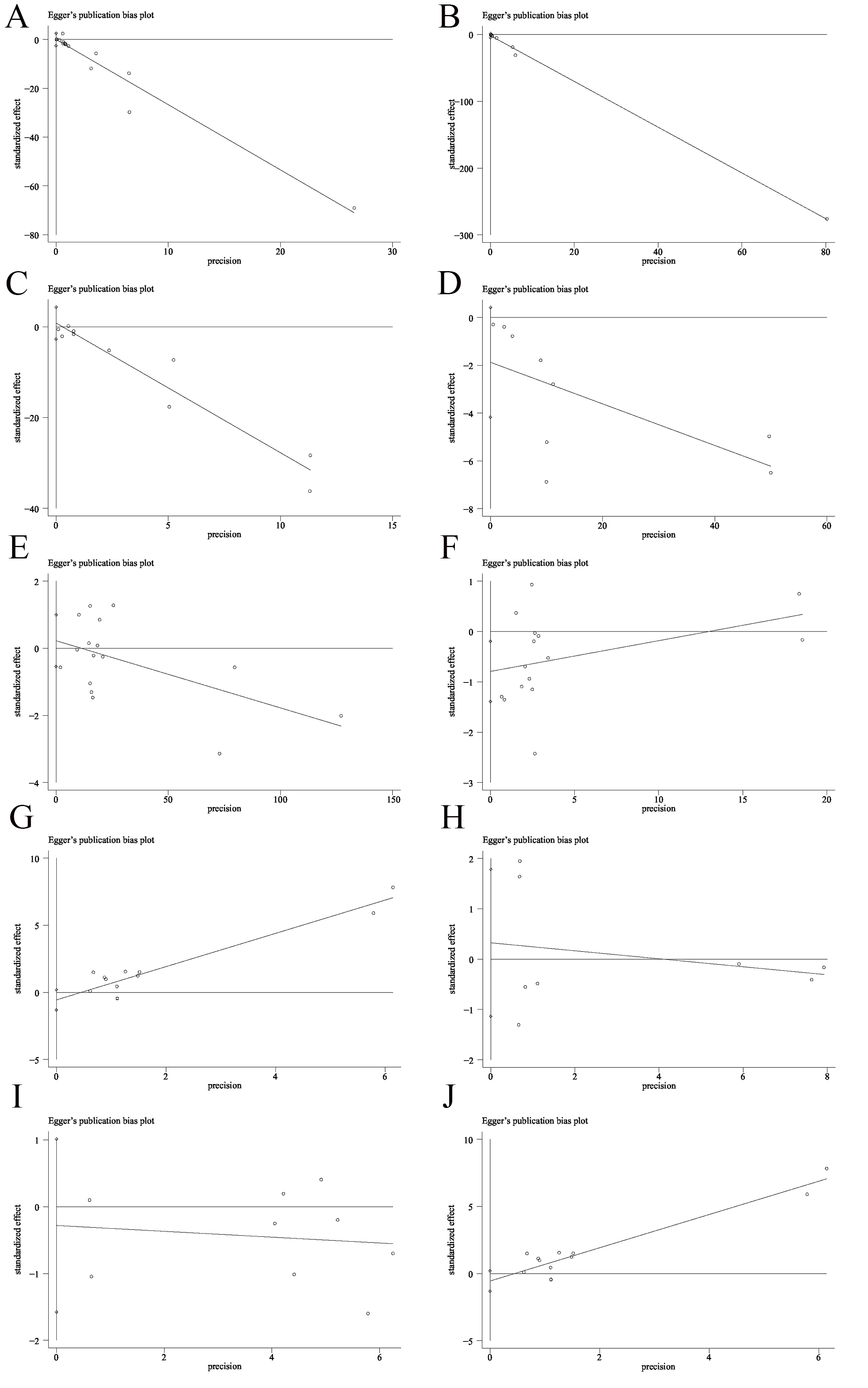

Supplement: Supplementary Figure 1 — Egger’s plots of eGFR (A), SBP (B), DBP (C), HbA1c (D), AEs (E), urinary tract infection (F), genital infection (G), bone fracture (H), hypoglycemia (I), and diabetic ketoacidosis (J). AEs, adverse events; DBP, diastolic blood pressure; eGFR, estimated glomerular filtration rate; SBP, systolic blood pressure. [file Image1.tif]

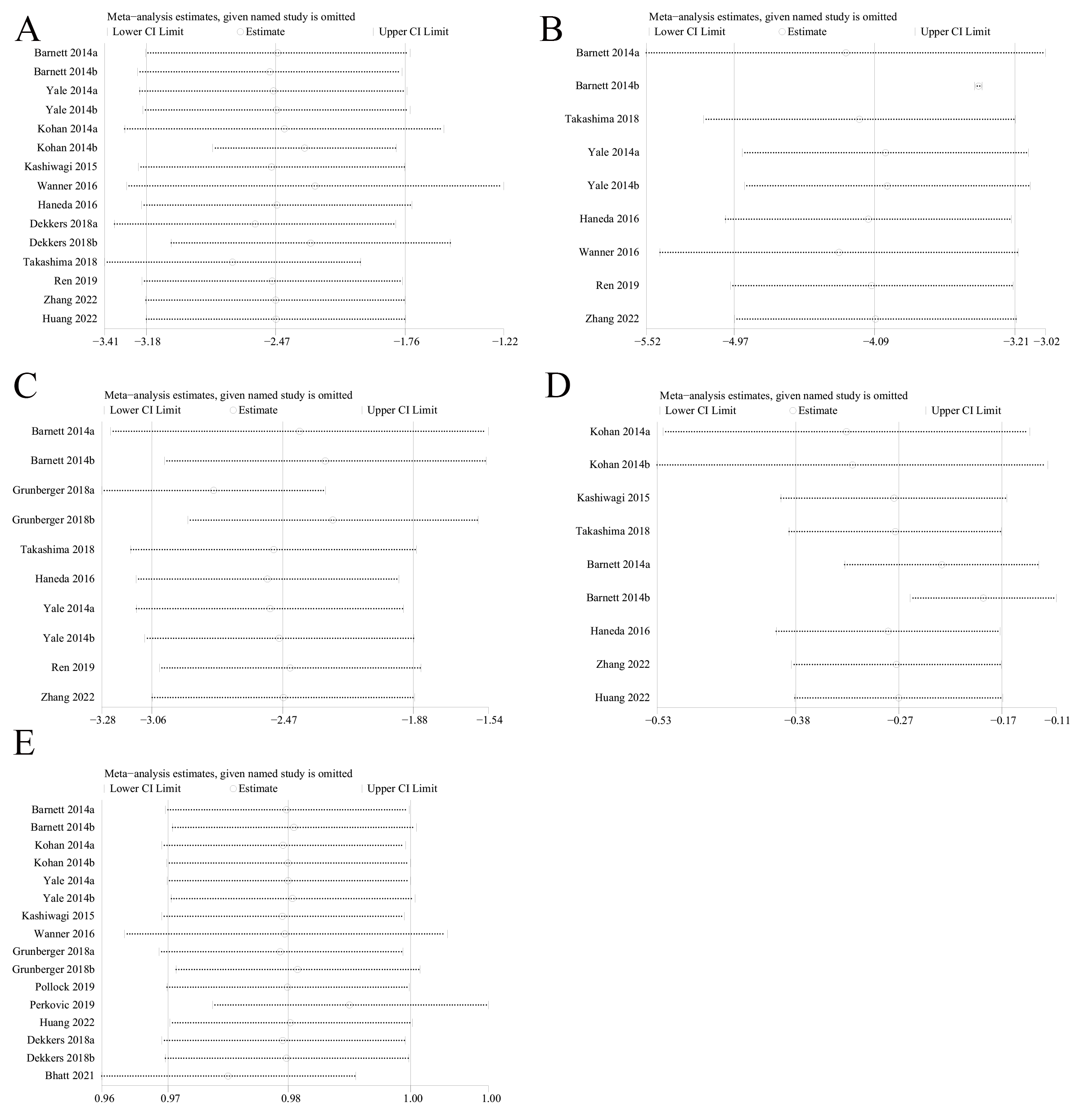

Supplement: Supplementary Figure 2 — Sensitivity analysis of eGFR (A), SBP (B), DBP (C), HbA1c (D), and AEs (E). [file Image2.tif]
