# Supplementary material for: Efficacy and safety of sodium-glucose cotransporter 2 inhibitors in the treatment of diabetic kidney disease: a meta-analysis
Source: Front Endocrinol (Lausanne). 2026 Jan 27;16:1596888. doi: 10.3389/fendo.2025.1596888 (PMC12886043; doi:10.3389/fendo.2025.1596888)
Supplement: Supplementary file 3 [file DataSheet1.docx]

**Supplementary Material 1. Detailed search strategy.**

**Pubmed:**

(((((((((((Diabetes Mellitus[Title/Abstract]) OR (Diabetes Insipidus[Title/Abstract])) OR (Diet, Diabetic[Title/Abstract])) OR (Prediabetic State[Title/Abstract])) OR (Scleredema Adultorum[Title/Abstract])) OR (Glycation End Products, Advanced[Title/Abstract])) OR (Glucose Intolerance[Title/Abstract])) OR (Gastroparesis[Title/Abstract])) OR (Diabetes[Title/Abstract])) AND ((((((((((((((((((((((Renal Insufficiency, Chronic[Title/Abstract]) OR (Chronic Renal Insufficiencies[Title/Abstract])) OR (Renal Insufficiencies, Chronic[Title/Abstract])) OR (Chronic Renal Insufficiency[Title/Abstract])) OR (Kidney Insufficiency, Chronic[Title/Abstract])) OR (Chronic Kidney Insufficiency[Title/Abstract])) OR (Chronic Kidney Insufficiencies[Title/Abstract])) OR (Kidney Insufficiencies, Chronic[Title/Abstract])) OR (Chronic Kidney Diseases[Title/Abstract])) OR (Chronic Kidney Disease[Title/Abstract])) OR (Disease, Chronic Kidney[Title/Abstract])) OR (Diseases, Chronic Kidney[Title/Abstract])) OR (Kidney Disease, Chronic[Title/Abstract])) OR (Kidney Diseases, Chronic[Title/Abstract])) OR (Chronic Renal Diseases[Title/Abstract])) OR (Chronic Renal Disease[Title/Abstract])) OR (Disease, Chronic Renal[Title/Abstract])) OR (Diseases, Chronic Renal[Title/Abstract])) OR (Renal Disease, Chronic[Title/Abstract])) OR (Renal Diseases, Chronic[Title/Abstract])) OR (CKD2[Title/Abstract])) OR (CKD3[Title/Abstract]))) OR ((((((((((((((((((Diabetic Nephropathies[Title/Abstract]) OR (Nephropathies, Diabetic[Title/Abstract])) OR (Nephropathy, Diabetic[Title/Abstract])) OR (Diabetic Nephropathy[Title/Abstract])) OR (Diabetic Kidney Disease[Title/Abstract])) OR (Diabetic Kidney Diseases[Title/Abstract])) OR (Kidney Disease, Diabetic[Title/Abstract])) OR (Kidney Diseases, Diabetic[Title/Abstract])) OR (Diabetic Glomerulosclerosis[Title/Abstract])) OR (Glomerulosclerosis, Diabetic[Title/Abstract])) OR (Intracapillary Glomerulosclerosis[Title/Abstract])) OR (Nodular Glomerulosclerosis[Title/Abstract])) OR (Glomerulosclerosis, Nodular[Title/Abstract])) OR (Kimmelstiel-Wilson Syndrome[Title/Abstract])) OR (Kimmelstiel Wilson Syndrome[Title/Abstract])) OR (Syndrome, Kimmelstiel-Wilson[Title/Abstract])) OR (Kimmelstiel-Wilson Disease[Title/Abstract])) OR (Kimmelstiel Wilson Disease[Title/Abstract]))) AND (((((((((((((((((((((((((((((((((((((SGLT2 inhibitor[Title/Abstract]) OR (Sodium-glucose cotransporter 2 inhibitor[Title/Abstract])) OR (canagliflozin[Title/Abstract])) OR (Invokana[Title/Abstract])) OR (Canagliflozin Hemihydrate[Title/Abstract])) OR (Canagliflozin, Anhydrous[Title/Abstract])) OR (dapagliflozin[Title/Abstract])) OR (Farxiga[Title/Abstract])) OR (Forxiga[Title/Abstract])) OR (BMS 512148[Title/Abstract])) OR (BMS512148[Title/Abstract])) OR (BMS-512148[Title/Abstract])) OR (empagliflozin[Title/Abstract])) OR (BI 10773[Title/Abstract])) OR (BI10773[Title/Abstract])) OR (BI-10773[Title/Abstract])) OR (Jardiance[Title/Abstract])) OR (Ipragliflozin[Title/Abstract])) OR (Suglat[Title/Abstract])) OR (ASP1941[Title/Abstract])) OR (ASP-1941[Title/Abstract])) OR (luseogliflozin[Title/Abstract])) OR (Lusefi[Title/Abstract])) OR (TS 071[Title/Abstract])) OR (TS071 cpd[Title/Abstract])) OR (TS-071[Title/Abstract])) OR (Ertugliflozin[Title/Abstract])) OR (Steglatro[Title/Abstract])) OR (PF 04971729[Title/Abstract])) OR (PF04971729[Title/Abstract])) OR (PF-04971729[Title/Abstract])) OR (tofogliflozin hydrate[Title/Abstract])) OR (CSG452[Title/Abstract])) OR (tofogliflozin anhydrous[Title/Abstract])) OR (Apleway[Title/Abstract])) OR (Deberza[Title/Abstract])) OR (tofogliflozin[Title/Abstract])) Filters: **Randomized Controlled Trial**

**Web of science:**

(((Diabetes Mellitus) OR (Diabetes Insipidus) OR (Diet, Diabetic) OR (Prediabetic State) OR (Scleredema Adultorum) OR (Glycation End Products, Advanced) OR (Glucose Intolerance) OR (Gastroparesis) OR (Diabetes)) AND (((Renal Insufficiency, Chronic) OR (Chronic Renal Insufficiencies) OR (Renal Insufficiencies, Chronic) OR (Chronic Renal Insufficiency) OR (Kidney Insufficiency, Chronic) OR (Chronic Kidney Insufficiency) OR (Chronic Kidney Insufficiencies) OR (Kidney Insufficiencies, Chronic) OR (Chronic Kidney Diseases) OR (Chronic Kidney Disease) OR (Disease, Chronic Kidney) OR (Diseases, Chronic Kidney) OR (Kidney Disease, Chronic) OR (Kidney Diseases, Chronic) OR (Chronic Renal Diseases) OR (Chronic Renal Disease) OR (Disease, Chronic Renal) OR (Diseases, Chronic Renal) OR (Renal Disease, Chronic) OR (Renal Diseases, Chronic) OR (CKD2) OR (CKD3)) OR ((Diabetic Nephropathies) OR (Nephropathies, Diabetic) OR (Nephropathy, Diabetic) OR (Diabetic Nephropathy) OR (Diabetic Kidney Disease) OR (Diabetic Kidney Diseases) OR (Kidney Disease, Diabetic) OR (Kidney Diseases, Diabetic) OR (Diabetic Glomerulosclerosis) OR (Glomerulosclerosis, Diabetic) OR (Intracapillary Glomerulosclerosis) OR (Nodular Glomerulosclerosis) OR (Glomerulosclerosis, Nodular) OR (Kimmelstiel-Wilson Syndrome) OR (Kimmelstiel Wilson Syndrome) OR (Syndrome, Kimmelstiel-Wilson) OR (Kimmelstiel-Wilson Disease) OR (Kimmelstiel Wilson Disease)))) AND ((SGLT2 inhibitor) OR (Sodium-glucose cotransporter 2 inhibitor) OR (canagliflozin) OR (Invokana) OR (Canagliflozin Hemihydrate) OR (Canagliflozin, Anhydrous) OR (dapagliflozin) OR (Farxiga) OR (Forxiga) OR (BMS 512148) OR (BMS512148) OR (BMS-512148) OR (empagliflozin) OR (BI 10773) OR (BI10773) OR (BI-10773) OR (Jardiance) OR (Ipragliflozin) OR (Suglat) OR (ASP1941) OR (ASP-1941) OR (luseogliflozin) OR (Lusefi) OR (TS 071) OR (TS071 cpd) OR (TS-071) OR (Ertugliflozin) OR (Steglatro) OR (PF 04971729) OR (PF04971729) OR (PF-04971729) OR (tofogliflozin hydrate) OR (CSG452) OR (tofogliflozin anhydrous) OR (Apleway) OR (Deberza) OR (tofogliflozin)) AND ((Randomized clinical trial) OR (RCT) OR (RCTs) OR (Clinical Trials) OR (Clinical controlled trial))

**Embase:**

((‘Diabetes Mellitus’:ab,ti OR ‘Diabetes Insipidus’:ab,ti OR ‘Diet, Diabetic’:ab,ti OR ‘Prediabetic State’:ab,ti OR ‘Scleredema Adultorum’:ab,ti OR ‘Glycation End Products, Advanced’:ab,ti OR ‘Glucose Intolerance’:ab,ti OR ‘Gastroparesis’:ab,ti OR ‘Diabetes’:ab,ti) AND ((‘Renal Insufficiency, Chronic’:ab,ti OR ‘Chronic Renal Insufficiencies’:ab,ti OR ‘Renal Insufficiencies, Chronic’:ab,ti OR ‘Chronic Renal Insufficiency’:ab,ti OR ‘Kidney Insufficiency, Chronic’:ab,ti OR ‘Chronic Kidney Insufficiency’:ab,ti OR ‘Chronic Kidney Insufficiencies’:ab,ti OR ‘Kidney Insufficiencies, Chronic’:ab,ti OR ‘Chronic Kidney Diseases’:ab,ti OR ‘Chronic Kidney Disease’:ab,ti OR ‘Disease, Chronic Kidney’:ab,ti OR ‘Diseases, Chronic Kidney’:ab,ti OR ‘Kidney Disease, Chronic’:ab,ti OR ‘Kidney Diseases, Chronic’:ab,ti OR ‘Chronic Renal Diseases’:ab,ti OR ‘Chronic Renal Disease’:ab,ti OR ‘Disease, Chronic Renal’:ab,ti OR ‘Diseases, Chronic Renal’:ab,ti OR ‘Renal Disease, Chronic’:ab,ti OR ‘Renal Diseases, Chronic’:ab,ti OR ‘CKD2’:ab,ti OR ‘CKD3’:ab,ti) OR (‘Diabetic Nephropathies’:ab,ti OR ‘Nephropathies, Diabetic’:ab,ti OR ‘Nephropathy, Diabetic’:ab,ti OR ‘Diabetic Nephropathy’:ab,ti OR ‘Diabetic Kidney Disease’:ab,ti OR ‘Diabetic Kidney Diseases’:ab,ti OR ‘Kidney Disease, Diabetic’:ab,ti OR ‘Kidney Diseases, Diabetic’:ab,ti OR ‘Diabetic Glomerulosclerosis’:ab,ti OR ‘Glomerulosclerosis, Diabetic’:ab,ti OR ‘Intracapillary Glomerulosclerosis’:ab,ti OR ‘Nodular Glomerulosclerosis’:ab,ti OR ‘Glomerulosclerosis, Nodular’:ab,ti OR ‘Kimmelstiel-Wilson Syndrome’:ab,ti OR ‘Kimmelstiel Wilson Syndrome’:ab,ti OR ‘Syndrome, Kimmelstiel-Wilson’:ab,ti OR ‘Kimmelstiel-Wilson Disease’:ab,ti OR ‘Kimmelstiel Wilson Disease’:ab,ti))) AND (‘SGLT2 inhibitor’:ab,ti OR ‘Sodium-glucose cotransporter 2 inhibitor’:ab,ti OR ‘canagliflozin’:ab,ti OR ‘Invokana’:ab,ti OR ‘Canagliflozin Hemihydrate’:ab,ti OR ‘Canagliflozin, Anhydrous’:ab,ti OR ‘dapagliflozin’:ab,ti OR ‘Farxiga’:ab,ti OR ‘Forxiga’:ab,ti OR ‘BMS 512148’:ab,ti OR ‘BMS512148’:ab,ti OR ‘BMS-512148’:ab,ti OR ‘empagliflozin’:ab,ti OR ‘BI 10773’:ab,ti OR ‘BI10773’:ab,ti OR ‘BI-10773’:ab,ti OR ‘Jardiance’:ab,ti OR ‘Ipragliflozin’:ab,ti OR ‘Suglat’:ab,ti OR ‘ASP1941’:ab,ti OR ‘ASP-1941’:ab,ti OR ‘luseogliflozin’:ab,ti OR ‘Lusefi’:ab,ti OR ‘TS 071’:ab,ti OR ‘TS071 cpd’:ab,ti OR ‘TS-071’:ab,ti OR ‘Ertugliflozin’:ab,ti OR ‘Steglatro’:ab,ti OR ‘PF 04971729’:ab,ti OR ‘PF04971729’:ab,ti OR ‘PF-04971729’:ab,ti OR ‘tofogliflozin hydrate’:ab,ti OR ‘CSG452’:ab,ti OR ‘tofogliflozin anhydrous’:ab,ti OR ‘Apleway’:ab,ti OR ‘Deberza’:ab,ti OR ‘tofogliflozin’:ab,ti) AND (‘Randomized clinical trial’:ab,ti OR ‘RCT’:ab,ti OR ‘RCTs’:ab,ti OR ‘Clinical Trials’:ab,ti OR ‘Clinical controlled trial’:ab,ti)
